# Knockdown of miR‐423‐5p simultaneously upgrades the eNOS and VEGFa pathways in ADSCs and improves erectile function in diabetic rats

**DOI:** 10.1111/jcmm.16927

**Published:** 2021-09-20

**Authors:** Jun Zhou, Yinghao Yin, Yuan Yang, Dongyi Peng, Jingchao Wei, Guangming Yin, Yuxin Tang

**Affiliations:** ^1^ Department of Urology The Third Xiangya Hospital of Central South University Changsha China; ^2^ Department of Urology The Fifth Affiliated Hospital of Sun Yat‐sen University Zhuhai China; ^3^ Guangdong Provincial Key Laboratory of Biomedical Imaging The Fifth Affiliated Hospital Sun Yat‐sen University Zhuhai Guangdong Province China

**Keywords:** ADSCs, diabetes mellitus, eNOS, erectile dysfunction, miR‐423‐5p, VEGFa

## Abstract

This study aimed to explore the possibility of miR‐423‐5p modified adipose‐derived stem cell (ADSCs) therapy on streptozotocin (STZ)‐induced diabetes mellitus erectile dysfunction (DMED) rats. MiR‐423‐5p was knocked down in ADSCs. ADSCs, NC‐miR‐ADSCs and miR‐ADSCs were co‐cultured with human umbilical vein endothelial cells (HUVECs). Normal and high glucose media were supplemented. The supernatant and HUVECs were collected for assessment of eNOS and VEGFa expression, cell proliferation, and apoptosis. HUVECs co‐cultured with ADSCs or miR‐ADSCs exhibited higher eNOS and VEGFa protein expression levels compared to DM groups. MiR‐ADSCs enhanced HUVEC proliferation compared to the ADSCs and NC‐miR‐ADSCs. Lower apoptotic rates were observed when HUVECs were co‐cultured with miR‐ADSCs, compared to ADSCs and NC‐miR‐ADSCs. Fifteen male Sprague‐Dawley (SD) rats aged 12 weeks were induced to develop diabetes mellitus by intraperitoneal injection with STZ, and five healthy SD rats were used as normal controls. Eight weeks after developing diabetes, the rats received ADSCs and miR‐ADSCs via injection into the corpora cavernosa, whereas normal controls and DM controls were injected with saline. Erectile function and histological assessment of penile tissues were performed 8 weeks after injection. The ICP/MAP indicated that erectile function was impaired in the DM rats compared with the normal group. Injection of ADSCs and miR‐ADSCs improved erectile function significantly and was associated with the overexpression of eNOS and VEGFa. MiR‐423‐5p knockdown in ADSCs ameliorated high glucose‐mediated damage to HUVECs and improved erectile function in DM rats by inducing eNOS and VEGFa overexpression, indicating that miR‐423‐5p may be a potential target in the treatment of DMED.

## 1 BACKGROUND

The morbidity and mortality of diabetes mellitus (DM), which is associated with chronic complications, are continuously increasing.^1^, ^2^ Erectile dysfunction (ED) is more likely to happen in men with DM.^3^ In different populations and ages, the morbidity rate of ED among men with DM varies from 35% to 90%.^4^, ^5^ Diabetes mellitus‐induced erectile dysfunction (DMED), which has pathogenic features that include endothelial, neuropathic and microvascular damage and fibrous‐muscular alterations, is usually more severe and difficult to treat than nondiabetics, in disregard of the heavy burden.^6^, ^7^

Recently, several experimental approaches for DMED have been developed, including insulin treatment,^8^ antioxidant therapy,^9^ low energy shockwave therapy,^10^ stem cells and gene therapy.^11^, ^12^ Among these strategies, stem cell‐based therapy is considered promising due to its ability to recover functional cells and tissues. In choosing the candidate stem cells from many kinds of mesenchymal stem cells, adipose‐derived stem cells (ADSCs) appear to be one of the most suitable types.^3^, ^13^ ADSCs have several biological benefits, including a large amount of autologous sources, ease in isolation and the ability to expand.^14^ The results of studies in rats with diabetes and cavernous injury, which were treated with the intracavernous injection of ADSCs, showed that erectile function had been restored.^15^, ^16^, ^17^

Endothelial nitric oxide synthase (eNOS) plays a key role in penile erection. The blood flow‐induced phosphatidylinositol 3‐kinase/Akt/eNOS phosphorylation cascade reducing the calcium dependence and sustaining endothelial nitric oxide (NO) release and making the cavernous smooth muscle relax continuously, helps the penile erection maintenance.^18^ Endothelial and smooth muscle cells can secrete vascular endothelial growth factor (VEGF) which is a multifunctional glycoprotein. Receptor‐mediated endothelial proliferation can be induced by VEGF in vitro and in vivo. And VEGF is related to endothelial function and an effective vasculogenic and vascular permeability factor.^19^ Recently, studies have shown that endogenous NO production can be stimulated by VEGF, which can play positive roles on endothelial and smooth muscle cells, resulting in improvement in erectile function.^20^, ^21^

MicroRNAs (miRs) are short non‐coding RNA that are 18–23 nucleotides in length.^22^ miRs function in regulating gene expression by binding to the 3’ untranslated region of the target mRNA.^23^ In this study, bioinformatics analysis indicated that miR‐423‐5p has the ability to regulate both eNOS and VEGFa genes. Thus, we investigated whether miR‐423‐5p can improve endothelial cell function and ameliorate erectile function in DM rats, as both eNOS and VEGFa genes play key functions in the mechanism of penis erection. To the best of our knowledge, this is the first study to investigate miRs regulating two important genes in a DMED study.

## 2 MATERIALS AND METHODS

### 2.1 Cell culture

Adipose‐derived stem cells (ADSCs) and HUVECs were purchased from Procell Life. ADSCs were maintained in stem cell culture medium supplemented with 10% fetal bovine serum and antibiotics (100 units/ml penicillin and 100 mg/ml streptomycin). HUVECs were maintained in endothelial cell basal medium supplemented with 1% endothelial cell growth supplement and 10% fetal bovine serum. The glucose concentration of the high glucose medium was 30 mmol/L. The ADSCs were transfected with CRISPR lentivirus‐carrying miR‐423‐5p or miR‐negative control (miR‐NC) (Shanghai GenePharma Co., Ltd). The sequence of gRNA is‘gNNNNNNNNNNNNNNNNNNNNgttttagagctaGAAAtagcaagttaaaataaggctagtccgttatcaacttgaaaaagtggcaccgagtcggtgcTTT’. ADSCs, miR‐NC‐ADSCs and miR‐ADSCs were co‐cultured with HUVECs, and the supernatant was collected for enzyme‐linked immunosorbent assay (ELISA).

### 2.2 Animals

Experiments were approved by the institute of Ethics Committee of the third Xiangya Hospital of Central South University. Forty male Sprague‐Dawley (SD) rats were acquired from animal center of Central South University. Rats in DM groups were intraperitoneally injected with STZ (60 mg kg^−1^; Sigma‐Aldrich) after fasting for 16 h. Fasting blood glucose levels were measured at 3 days after STZ injection using a blood glucose meter. Fasting glucose concentration higher than 16 mmol/L was considered as a success of DM. At 8 weeks after STZ injection, apomorphine (100 μg kg^−1^; Sigma‐Aldrich) was used to screen the diabetic rats. Then the DMED rats were randomly divided into three groups (five in each group): DMED control, ADSCs + DMED, miR‐423‐5p‐ADSCs + DMED. And five normal rats with an IC injection of PBS were set as a normal control group. Under aseptic conditions, the determined DMED and normal rats were anaesthetized with 3% diethyl ether. The penis was exposed in each group, and a 24‐gauge needle was used to inject a total of 1 × 106 ADSCs or miR‐423‐5p‐ADSCs in 100 μl PBS or only 100 μl PBS into the corpus cavernosum.

### 2.3 Cell apoptosis analysis

Apoptosis was assessed with an Annexin V‐FITC/PI Apoptosis Detection Kit (Beyotime Institute of Biotechnology) according to the manufacturer's instructions. Briefly, after treatment and incubation for 48 h, the cells were collected, washed with PBS and stained with Annexin V and propidium iodide (PtdIns) in the dark using an Annexin V‐FITC apoptosis detection kit (Beyotime Biotechnology). Cell apoptosis was subsequently analysed by FACSCalibur flow cytometry (BD Biosciences).

### 2.4 Quantitative real‐time PCR

Total RNA was extracted from tissue samples or cells using TRIzol following the manufacturer's instructions. Total RNA was reverse transcribed into cDNA using a PrimeScript RT reagent kit. Reverse transcription‐polymerase chain reaction (RT‐PCR) was performed using the iQ5 Multicolor Real‐Time PCR Detection System (Bio‐Rad Laboratories, Inc.) with SYBR Premix Ex Taq II. The PCR primer sequences for miR‐423‐5p and U6snRNA were as follows: miR‐423‐5p forward primer 5′‐ACACTCCAGCTGGGTGAGGGGCAGAGAGCGA‐3′, reverse primer 5′‐CTCAACTGGTGTCGTGGAGTCGGCAATTCAGTTGAGAAAGTCTC‐3′; U6snRNA forward primer 5′‐CTCGCTTCGGCAGCACA‐3′ and reverse primer 5′‐AACGCTTCACGAATTTGCGT‐3′. A melting curve analysis of the amplified products was performed at the end of each PCR cycle. U6snRNA was used as internal control, and gene expression was relatively quantified using the 2‐∆∆CT method.

### 2.5 Western blotting

Western blotting (WB) was performed as described previously. Briefly, the cells and rat penises were lysed in lysis buffer containing protease inhibitors. Protein concentrations of the lysates were determined by the bicinchoninic acid assay (Beyotime Biotechnology). Equal amounts of protein (20 µg) were separated by 10% SDS‐PAGE and subsequently transferred onto PVDF membranes. The membranes were blocked with 5% non‐fat dry milk in 0.2% Tween‐20 in Tris‐buffered saline (TBS‐T) for 1 h at room temperature and then hybridized with primary antibodies. The primary antibodies were mouse anti‐eNOS (1:400), mouse anti‐VEGFa (1:400) and mouse anti‐glyceraldehyde phosphate dehydrogenase (GAPDH, 1:10,000). Immunoreactivity was detected after incubation with a horseradish peroxidase‐conjugated secondary antibody according to the manufacturer's instructions (Thermo Scientific). GAPDH was used as a loading control. The positive bands were analysed using Gel‐pro analyzer software, and integrated optical density (IOD) was measured.

### 2.6 ELISA

The cell culture medium was collected after coculture, the supernatant was collected by centrifugation for 10 min at 1500 rpm. eNOS and VEGFa expression levels were measured using an ELISA kit (Solarbio Life Sciences) according to the manufacturer's instructions (SEA868Ra for eNOS, SEA143Ra for VEGFa). Absorption at a wavelength of 450 nm (A450) was determined using the microplate reader.

### 2.7 Masson trichrome stain

Masson trichrome staining using a Trichrome Stain (Masson) Kit (Sigma‐Aldrich Co.) was performed to visualize fibers in tissues, following the manufacturer's instructions. Briefly, the tissue slides were deparaffinized, stained in preheated Bouin's solution, and washed in running tap water to remove the yellow colour from sections. Then, the slides were respectively stained in Working Weigert's Iron Hematoxylin Solution, Biebrich Scarlet‐Acid Fucshin, Working Phosphotungstic/Phosphomolybdic Acid Solution and Aniline Blue Solution. The stained slides were observed under an optical microscope (magnification 40×).

### 2.8 Immunofluorescene staining

For immunofluorescene staining (IF), the primary antibodies were mouse anti‐eNOS (1:400), mouse anti‐VEGFa (1:400), secondary antibodies included Alexa‐488‐conjugated antibodies and Alexa‐592‐conjugated antibodies (1:500), nuclear staining was accomplished with 4’,6‐diamidino‐2‐phenylindole (DAPI).

### 2.9 Intracavernosal pressure (ICP) measurement

Erectile function was determined by intracavernosal pressure (ICP) and mean arterial pressure (MAP) 4 weeks post‐injection. Under 3% pentobarbital sodium, the major pelvic ganglion (MPG) and cavernous nerves (CN) were exposed by midline laparotomy. The penile was exposed by removing overlying skin and ischiocavernosus muscle. One of the 24‐gauge needles that were connected to PE‐50 tubes with heparinized saline (250 IU ml^−1^) was inserted into the left carotid to measure MAP. The other one was inserted into corpus cavernosum (CC) to measure ICP. PE‐50 tubes were connected to the data acquisition system (MP150, BIOPAC Systems Inc.). The CNs were stimulated using a stainless steel bipolar hook electrode with the following parameters: 20 Hz, pulse width of 0.2 ms, 1.5 mA, for 50 s. The ratio of maximal ICP (mm Hg) to MAP (mm Hg) was calculated.

### 2.10 Luciferase reporter assay

PmirGLO‐NOS3.3UTR and pmirGLO‐VEGFA.3UTR were constructed. The miR‐423‐5p mimics and negative control sequence were as follows: 5′‐UGAGGGGCAGAGAGCGAGACUUU‐3′ and 5′‐UUCUCCGAACGUGUCACGUTT‐3′. pRL‐TK vector (Takara Biotechnology Ltd.) of *Renilla* luciferase was used as internal reference for adjusting the differences in cell number and transfection efficiency. Approximately 2 × 10^4^ cells were seeded into a 48‐well plate individually and co‐transfected with 500 ng pmirGLO‐NOS3.3UTR and pmirGLO‐VEGFA.3UTR. Subsequently, the cells were transfected with mimics negative control and miR‐423‐5p mimics respectively. After 48 h of transfection, the luciferase assay was conducted using a dual luciferase reporter assay (Promega) according to the manufacturer's instructions.

### 2.11 Statistical analysis

All statistical analyses were performed with SPSS 19.0 (SPSS Inc.). All results are expressed as the mean ± standard deviation (SD). Multiple comparisons between groups were performed using ANOVA followed by post hoc analysis using the Tukey‐Kramer test, whereas a comparison between two groups was performed using a *t*‐test. Differences with a *p* < 0.05 were considered statistically significant.

## 3 RESULTS

### 3.1 MiR‐423‐5p directly targets eNOS and VEGFa mRNAs

**FIGURE 1:**
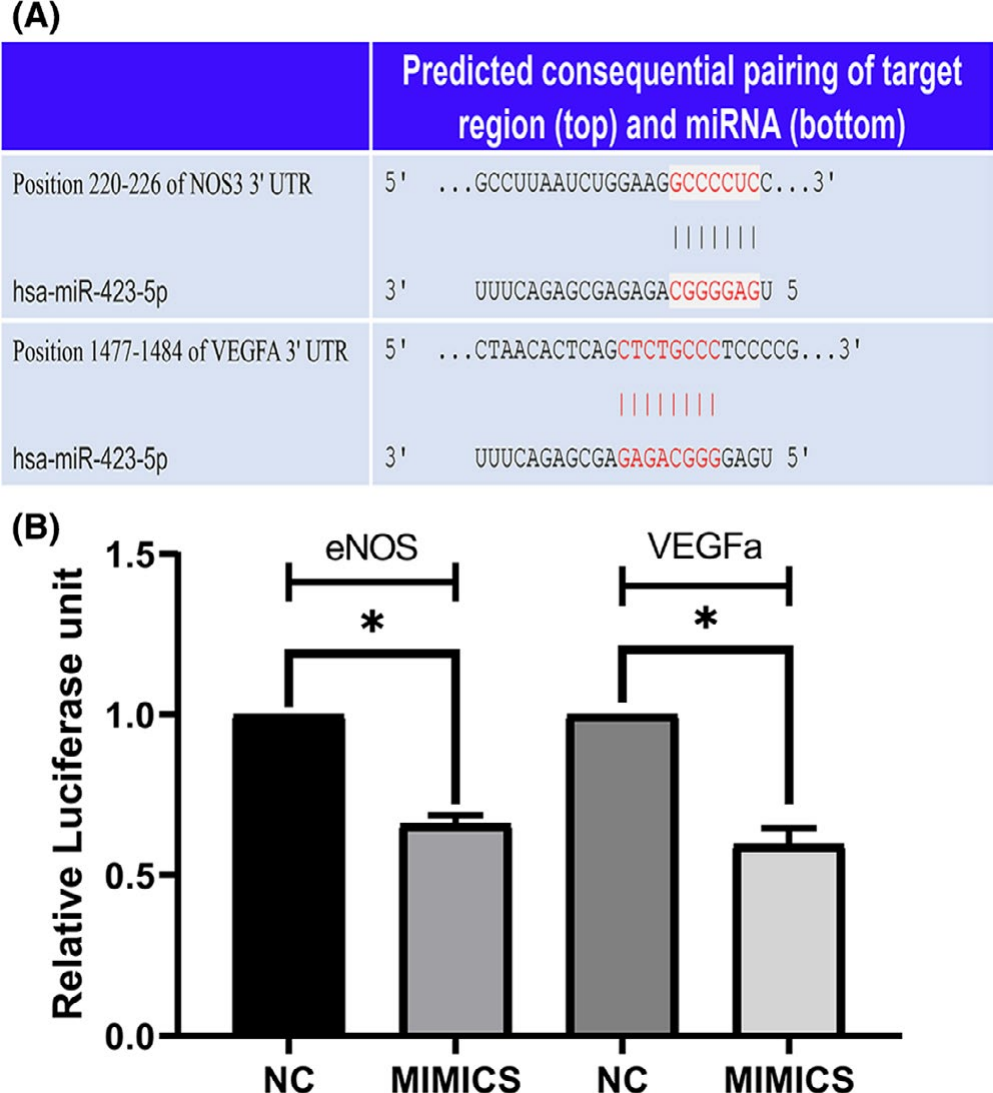
NOS3 and VEGFa are the target genes of miR‐423‐5p. (A) Predicted miR‐423‐5p binding sites of the NOS3 and VEGFa genes are shown in red. (B) Luciferase reporter assay showing post‐transcriptional repression of the NOS3 and VEGFa genes. Cells were cotransfected with miR‐423‐5p mimics and pmirGLO‐NOS3.3UTR or pmirGLO‐VEGFA.3UTR cloned into expression vector downstream of the luciferase gene

Bioinformatics prediction revealed that miR‐423‐5p has one specific potential binding site for eNOS and VEGFa mRNAs within the 3’‐UTR (Figure 1A). There are seven base‐pairs at the binding site for eNOS mRNA and miR‐423‐5p, and eight for VEGFa mRNA and miR‐423‐5P. A luciferase assay was performed to validate this prediction. Both eNOS and VEGFa luciferase activity were suppressed relative to the control; the suppression of eNOS was 66%, whereas VEGFa was 60% (Figure 1B). These findings indicate that eNOS and VEGFa are target genes of miR‐423‐5p.

### 3.2 miR‐423‐5p knockout improves eNOS and VEGFa protein expression in ADSCs

**FIGURE 2:**
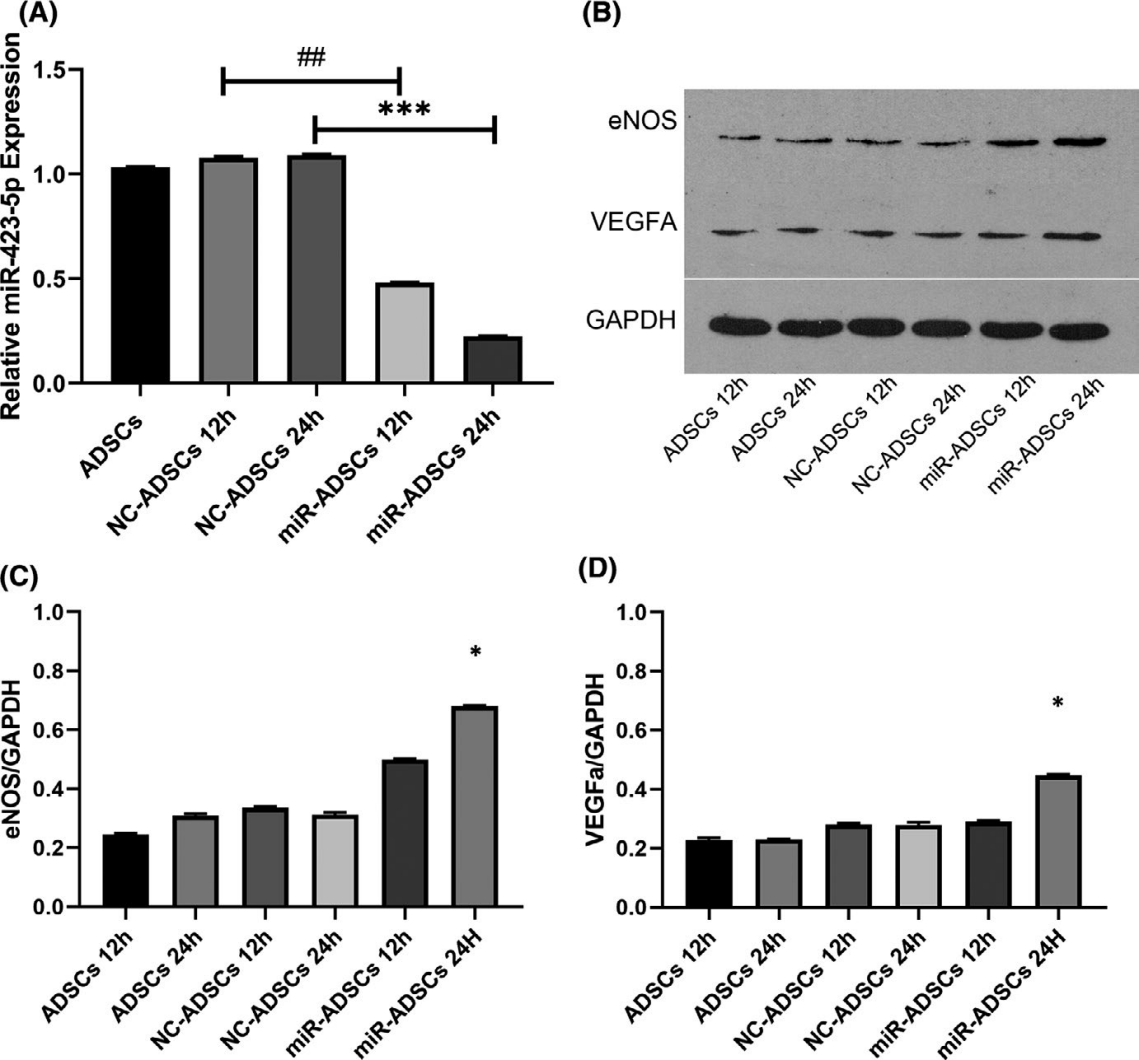
(A) Relative miR‐423‐5p expression after miR‐423‐5p knockdown in ADSCs. Approximately 12 and 24 h after inhibition of miR‐423‐5p, RNA was extracted for qRT‐PCR analysis. miR‐423‐5p expression was downregulated at both 12 and 24 h (*p* < 0.001). (B) Western blot: eNOS and VEGFA expression after miR‐423‐5p knockdown in ADSCs. (C and D) Relative eNOS and VEGFa expression levels increased with miR‐423‐5p inhibition. *p* < 0.05 versus normal ADSCs

To confirm whether miR‐423‐5p can influence eNOS and VEGFa protein expression, miR‐423‐5p was knocked down in ADSCs. miR‐423‐5p expression decreased after transfection with the knockout virus. At 12 and 24 h, miR‐423‐5p expression was markedly reduced (Figure 2A) (*p* < 0.001). To demonstrate the silencing effects of miR‐423‐5p on eNOS and VEGFa, total proteins in ADSCs were extracted for western blot analysis. Compared with the control group, eNOS and VEGFa expression levels significantly increased (Figure 2B–D) (*p* < 0.05). These results suggest that miR‐423‐5p inhibits protein expression of eNOS and VEGFa.

### 3.3 Knocking out miR‐423‐5p in ADSCs alleviates high glucose‐induced damage in HUVECs

**FIGURE 3:**
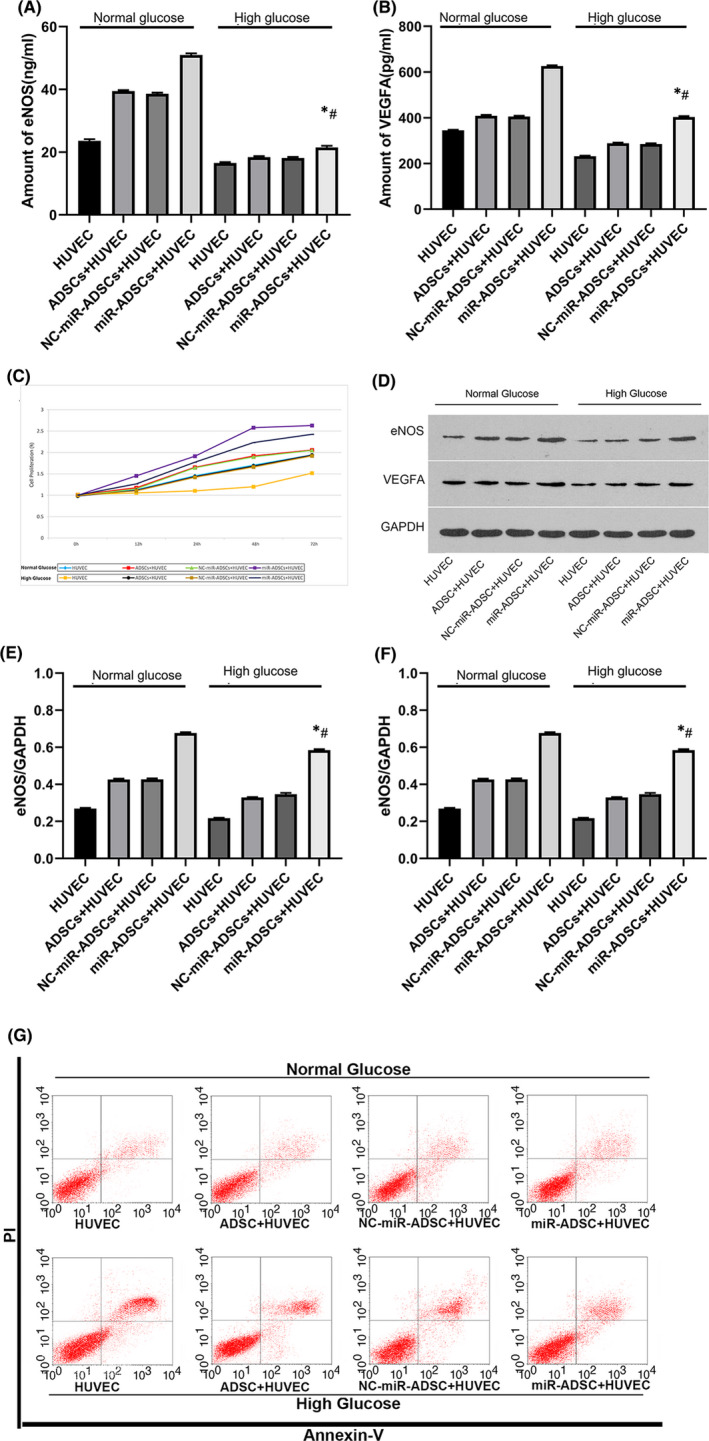
Changes in cell function when HUVECs are co‐cultured with ADSCs, NC‐miR‐ADSCs and miR‐ADSCs in normal or high glucose supplemented media. (A) eNOS expression in the culture medium; (B) VEGFa expression in the culture medium; (C) cell proliferation; (D) eNOS and VEGFa protein expression levels; (E) relative eNOS protein expression levels; (F) relative VEGFa protein expression levels; (G) flow cytometry; *y* axis is propidium Iodide(PI), *x* is Annexin V. The upper right quadrant represents late apoptotic and necrotic cells, while the lower right quadrant represents early apoptotic cells. *p < 0.01 compared to miR‐ADSCs + HUVEC in normal glucose conditions; ^#^ *p* < 0.05 compared to NC‐miR‐ADSCs + HUVEC in high glucose conditions

We transfected ADSCs with the miR‐423‐5p inhibition lentivirus, and then miR‐ADSCs were co‐cultured with HUVECs. In normal glucose conditions, ADSCs, NC‐miR‐ADSCs and miR‐ADSCs promoted eNOS and VEGFa expression in HUVEC conditioned media (Figure 3A,B). miR‐ADSCs increased the protein expression of eNOS and VEGFa under high glucose conditions compared to ADSCs and NC‐miR‐ADSCs (Figure 3A,B). A CCK‐8 assay was performed to assess cell proliferation. miR‐ADSCs promoted HUVEC proliferation under both normal and high glucose conditions (Figure 3C).

eNOS and VEGFa are key proteins in penis erection. Therefore, western blotting was performed to evaluate eNOS and VEGFa expression. miR‐423‐5p inhibition in ADSCs was associated with increased HUVEC eNOS and VEGFa expression. This increase was more profound under high glucose concentrations than under normal glucose concentrations (Figure 3D–F). To further investigate whether the inhibition of miR‐423‐5p in ADSCs would benefit HUVECs, cell flow cytometry was also conducted. Propidium iodide (PI) was used to assess cell apoptosis. The number of late apoptotic cells caused by high glucose damage sharply decreased when co‐cultured with miR‐ADSCs (Figure 3G).

### 3.4 Erectile function assessment

**FIGURE 4:**
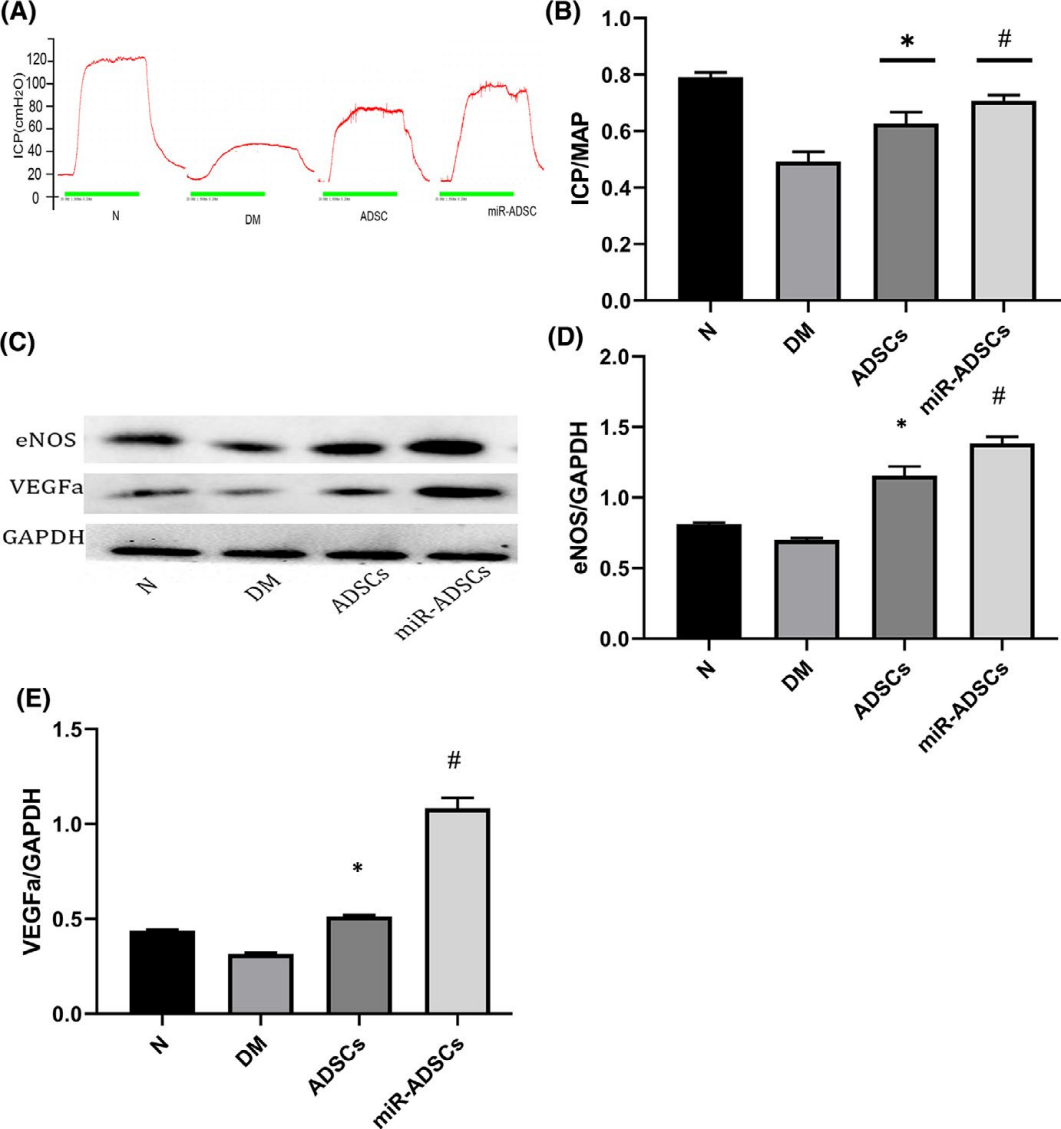
MiR‐ADSCs improve erectile function upon cavernous nerve electrostimulation in diabetic SD rats. (A) Representative tracings of Intracavernosal Pressure (ICP) in cm H_2_O for normal rats (N), diabetes mellitus rats (DM), ADSC‐injected rats (ADSCs) and miR‐ADSC‐injected rats (miR‐ADSC). (B) Erectile function is presented as the ICP/MAP ratio in each group. *p < 0.05 compared to the DM group; ^#^ *p* < 0.05 compared to the DM group. (C) eNOS and VEGFa protein expression levels; (D) relative eNOS protein expression levels, *p < 0.05 compared to the DM group, ^#^ *p* < 0.05 compared to the ADSC group; (E) relative VEGFa protein expression levels, *p < 0.05 compared to the DM group, ^#^ *p* < 0.05 compared to the ADSC group

**FIGURE 5:**
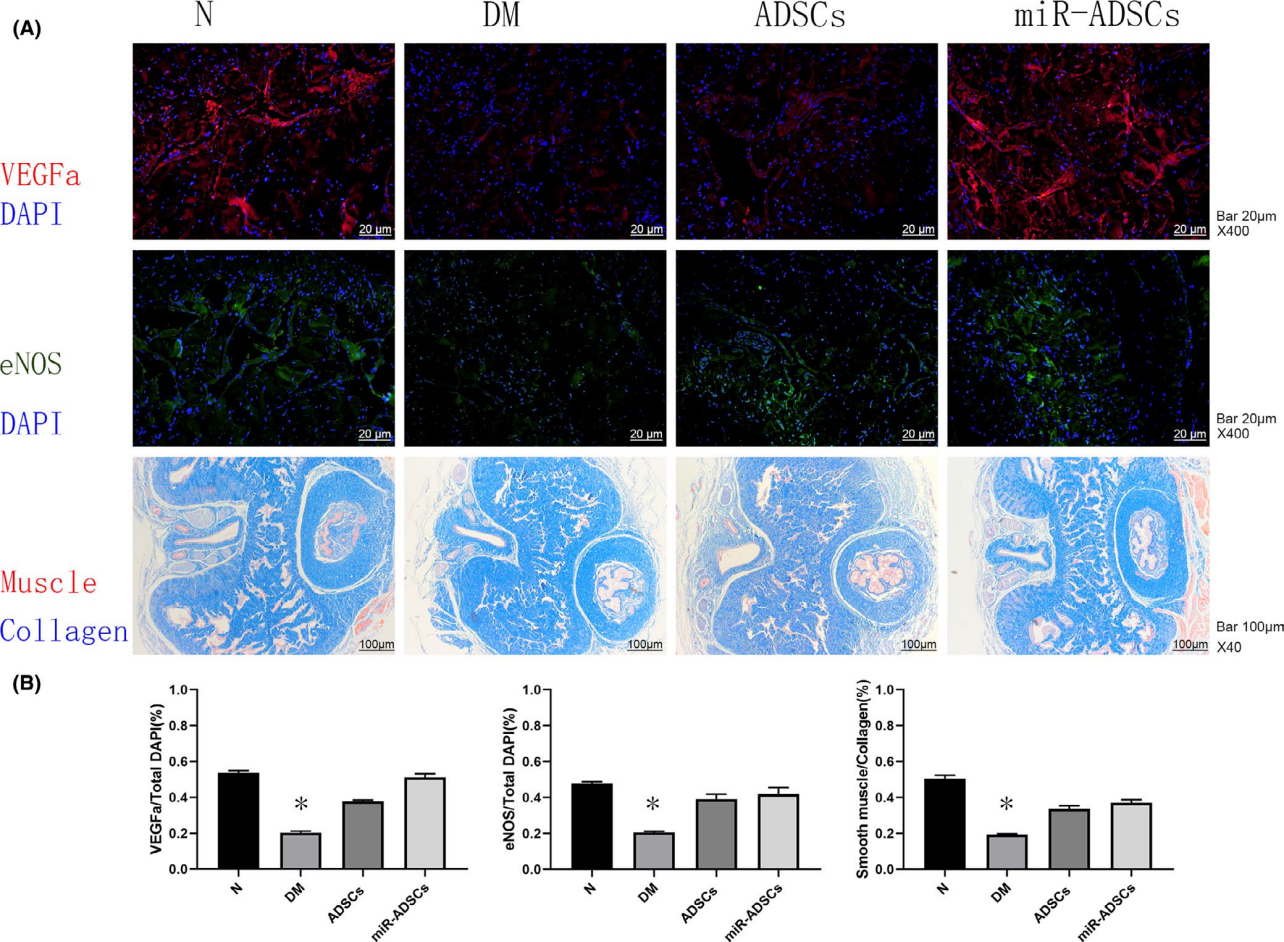
Expression of VEGFa and eNOS in the penile cavernous tissue 12 weeks after ADSCs/miR‐ADSCs implantation. (A) Expression of VEGFa and eNOS assessed by immunofluorescene staining and the mason trichrome staining of the cavernous tissue. (B) Comparison of VEGFa and eNOS expression and the ratio of muscle and collagen in different treatment groups. *p < 0.05 compared to the ADSCs group and miR‐ADSCs group

Figure 4A,B show that the DM group had lower ICP and ICP/MAP ratios than the ADSCs and miR‐ADSCs groups (*p* < 0.05). Interestingly, the miR‐ADSC group exhibited a significant increase in the ICP curve and ICP/MAP ratios compared to the ADSC group (*p* < 0.05). To assess eNOS and VEGFa expression, the penises from all rat groups were collected for western blotting analysis (Figure 4C–E) and immunofluorescene staining (Figure 5A,B), which showed that the DM group had lower eNOS and VEGFa protein expression than the ADSC or miR‐ADSC groups (*p* < 0.05). The ratio of smooth muscle:collagen staining, as determined by Masson's trichrome staining in penile tissues, was significantly lower in the DM group compared to the ADSC and miR‐ADSC groups (Figure 5). The ADSC and miR‐ADSC groups had a similar ratio of smooth muscle:collagen staining (Figure 5).

## 4 DISCUSSION

eNOS and VEGFa expression are both important for penile erection. Nitric oxide (NO) released from endothelium or nerve terminals stimulates the local production of cyclic guanosine monophosphate (cGMP), resulting in corporal smooth muscle relaxation, increased blood supply to the penis, and penile erection.^24^ Macrovasculopathy, microvasculopathy, and endothelial dysfunction are major factors that regulate blood flow in the penis.^3^ Microvascular disease and endothelial cell dysfunction play essential roles in DMED. In this study, a reduction in the number of smooth muscles in the corpus cavernosum was observed in diabetic rats. Moreover, diabetic rats showed a decrease in eNOS and VEGFa protein expression, compared to rats without diabetes.

Endothelial nitric oxide (eNOS) plays an essential role in penile erection. The neuronal NOS can release NO which can initiate the penile erection in a rapid, short‐term calcium‐dependent way. While, the blood flow‐induced phosphatidylinositol 3‐kinase/Akt/eNOS phosphorylation cascade make the full penile erection and maintenance of an erection. Sustained release of endothelial NO induces relaxation of local smooth muscle cells.^18^ Endothelial cells produce NO that helps maintain vascular integrity and enhance vasculogenesis.^25^ Previous studies have shown that eNOS‐based gene therapy restores impaired angiogenesis in rats^26^, ^27^ and that adenovirus‐mediated eNOS gene transfer can promote re‐endothelialization of blood vessel in injured rabbits.^28^

VEGFa is the most widely studied member of the VEGF family. Reduced VEGFa signaling and impaired angiogenesis as well as collateral blood vessel formation occur in patients with diabetes mellitus.^29^ Several groups have shown that humans and rats with ED have lower VEGFa expression than normal humans and rats, suggesting VEGFa could be a target for the treatment of ED. VEGFa also enhances the proliferation of cavernous smooth muscle cells and endothelial cells, further supporting erectile function.^30^ VEGFa can be induced by a NO synthesis pathway that also facilitates angiogenesis.^31^ Impaired vasculogenesis was reported in eNOS knockout mice (eNOS^−/−^).^32^ Diminished wound healing was also detected due to reduced VEGF‐mediated migration.^33^

ADSCs are somatic stem cells with multipotency and little immunogenicity.^34^ Moreover, ADSCs have been used to treat many diseases including the repair of muscular tissue.^35^ Several groups have investigated the feasibility and advantage of using ADSCs for ED therapy in rat models.^36^, ^37^ Implantation of ADSCs has been shown to significantly improve erection function in diabetic ED rats.^12^

MicroRNAs are small, non‐coding RNAs that modulate gene expression by binding to the 3’UTR of the target gene.^38^ MicroRNAs affect many biological processes, including cell apoptosis, proliferation and metabolism.^39^ A microRNA may not have a single target gene, instead influencing multiple genes simultaneously. We hereby describe an approach for improving eNOS and VEGFa expression for the potential treatment of DMED using microRNAs. MiR‐423‐5p can promote gluconeogenesis and hyperglycaemia.^40^ A computational biology study showed that miR‐423‐5p was at a high express level in the obesity and type 2 diabetes adipose tissue.^41^ Both hyperglycaemia and obesity are bad effects on erectile function.

In this study, we found that miR‐423‐5p simultaneously affected the expression of both eNOS and VEGFa. Knockout of miR‐423‐5p expression in ADSCs was associated with an increase in eNOS and VEGFa expression. ADSCs may differentiate into local smooth muscle cells or endothelial cells to restore organ function. ADSCs can also exert a local effect by secreting cytokines and growth factors.^17^, ^36^ In this study, ELISA and western blot results demonstrated that HUVEC co‐cultured with miR‐ADSCs showed overexpression of eNOS and VEGFa. As these two proteins play a key role in erection, we injected miR‐ADSCs and ADSCs into diabetes‐induced ED rats to evaluate their effect on erection. Both miR‐ADSC‐ and ADSC‐treated diabetic rats had improvement in erectile function. The miR‐ADSC‐treated group showed greater improvement than the ADSC‐treated group. Together, these findings indicate that knocking out miR‐423‐5p relieves the inhibition of eNOS and VEGFa expression in ADSCs, thereby supporting penile erection.

Our results were similar to previous studies in that we also used stem cells in treating DM rats. However, the present study has limitations, and our findings require further validation. We did not assess fibrosis factors that may cause tunica albuginea diseases such as Peyronie's disease. In addition, the mechanism by which ADSCs affect HUVECs warrants further investigation, including whether ADSCs secrete exosomes.

## 5 CONCLUSIONS

Knocking down miR‐423‐5p in ADSCs ameliorated the high glucose damage in HUVECs and improved erectile function in DM rats. There was an associated overexpression of eNOS and VEGFa, suggesting that miR‐423‐5p may be potentially used as a target in cell therapy for DM associated erectile dysfunction.

## CONFLICT OF INTEREST

All authors declare no competing financial interests.

## AUTHOR CONTRIBUTION

**Jun Zhou:** Conceptualization (lead); Investigation (lead); Writing‐original draft (equal); Writing‐review & editing (equal). **Yinghao Yin:** Methodology (equal). **Yuan Yang:** Data curation (supporting); Software (supporting). **Dongyi Peng:** Investigation (supporting). **Jingchao Wei:** Data curation (supporting); Software (supporting). **Guangming Yin:** Conceptualization (equal). **Yuxin Tang:** Funding acquisition (lead); Writing‐original draft (supporting); Writing‐review & editing (supporting).

## Data Availability

All datasets used during the current study are available from the corresponding author on reasonable request.
